# Preliminary Evaluation of Static and Kinematic Magnetic Resonance Imaging (MRI) of the Feline Temporomandibular Joint (TMJ): A Comparative Analysis of Joint Inclination Angles in Intact and Dislocated Conditions

**DOI:** 10.3390/vetsci13090956

**Published:** 2026-09-13

**Authors:** Basma Abdelmohsen, Francesca Del Signore, Martina Rosto, Elshaimaa Ismael, Fouad Farag, Massimo Vignoli

**Affiliations:** 1 Department of Anatomy and Embryology, Faculty of Veterinary Medicine, Cairo University, Giza 12613, Egypt; fouad.farag@cu.edu.eg; 2 Diagnostic Imaging Service, Faculty of Veterinary Medicine, University of Teramo, 64100 Teramo, Italy; mrosto@unite.it (M.R.); mvignoli@unite.it (M.V.); 3 Department of Veterinary Hygiene and Management, Faculty of Veterinary Medicine, Cairo University, Giza 12613, Egypt; elshaimaavet@cu.edu.eg

**Keywords:** TMJ, kMRI, static scans, inclination angles, felines

## Abstract

Conventional static MRI sequences routinely used in veterinary medicine provide limited information regarding joint motion and kinematics. Therefore, this study aimed to compare the diagnostic utility of static and kinematic MRI for the evaluation of the feline TMJ in cadavers and for the detection of TMJ dislocation using a low-field (0.25 T) MRI system. In addition, TMJ inclination angles were measured in both intact and experimentally dislocated joints at maximal interincisal opening (MIO) and in the closed-mouth position. Static MRI examinations were performed using T1-weighted (T1W), T2-weighted (T2W), and three-dimensional (3D) sequences acquired in both the closed-mouth and MIO positions. kMRI was performed using a 2D HYCE S sequence during repetitive mandibular opening and closing movements. Subsequently, TMJ dislocation was experimentally induced in a subset of cadavers, which were then rescanned using the same imaging protocol. Inclination angle measurements were analyzed using linear mixed-effects models. Compared with static MRI, kMRI demonstrated lower image quality, primarily due to motion artifacts and a reduced signal-to-noise ratio (SNR). TMJ inclination angles were significantly affected by both mouth position (*p* < 0.001) and dislocation status (*p* = 0.005). Mouth position had the greatest influence on inclination angles, with significantly larger values observed during maximal mouth opening than in the closed-mouth position. Furthermore, the left TMJ exhibited significantly greater inclination angles than the right TMJ (*p* = 0.012). Experimentally induced dislocation significantly altered TMJ inclination angles, indicating measurable biomechanical changes associated with joint instability.

## Simple Summary

This study investigated the morphological characteristics, MRI features, and biomechanics of the feline TMJ. The anatomical features of the feline TMJ were characterized using low-field MRI, providing detailed visualization of the joint structures. To evaluate joint biomechanics, feline cadavers were examined using kinematic MRI (kMRI), an imaging modality that has been rarely applied in veterinary medicine. Furthermore, a subset of feline cadavers was subjected to experimentally induced TMJ dislocation and subsequently evaluated as a model of joint instability. This approach was employed to assess the utility of MRI in detecting both intact and dislocated joints under static and kinematic imaging conditions. The results demonstrated that static MRI provided superior visualization of osseous and most soft tissue components except the articular disc and lateral ligament of the feline TMJ and reliably identified joint dislocation, enabling clear differentiation between normal and dislocated joints. In contrast, kMRI offered limited anatomical detail. These findings highlight the advantages of static low-field MRI for the assessment of feline TMJ anatomy and the diagnosis of joint dislocation.

## 1. Introduction

To date, several diagnostic imaging modalities have been used to evaluate the TMJ in both clinical and research settings in veterinary medicine. Among these, radiography (X-ray) and computed tomography (CT) remain the most used techniques for the assessment of TMJ disorders [1]. In contrast, MRI is considered the imaging modality of choice for evaluating both soft tissue and osseous components of the TMJ in human medicine because of its superior soft tissue contrast, multiplanar imaging capability, and ability to depict the complex anatomy of the joint and associated pathological conditions, including internal derangements [2,3,4]. In addition, it is considered the least invasive diagnostic modality with no risk of exposure to ionizing radiation. Furthermore, MRI provides excellent assessment of musculoskeletal abnormalities associated with neurological and orthopedic disorders [2].

Despite these advantages, the application of MRI in veterinary medicine remains limited due to relatively higher costs, long acquisition times, and the potential risks associated with anesthesia, particularly in compromised patients.

Evaluating the mechanics of the normal TMJ is of major importance in the diagnosis and treatment of injuries or disorders affecting the joints, as it determines the effectiveness of the treatment, as well as planning to develop surgical devices to restore joint function following trauma or disease [5]. Among TMJ disorders, TMJ luxation (dislocation) represents a serious clinical condition that may alter the biomechanics of the joint and is frequently associated with maxillofacial trauma. Previous studies have reported a higher incidence of TMJ luxation in cats than in dogs [6].

TMJ luxation most commonly occurs in either the rostral or caudal direction. Rostral luxation is typically characterized by mandibular deviation toward the unaffected side, whereas the comparatively uncommon caudal luxation results in deviation toward the affected side [7,8]. Clinical manifestations may include dysphagia, dental malocclusion, excessive salivation, pain or discomfort during mandibular movement, and an inability to properly open or close the mouth [6].

Earlier investigations explored TMJ morphology in cats [9], while biomechanics in cats and dogs have estimated the vertical mandibular range of motion in anesthetized animals [1]. Additionally, CT-based studies have documented the normal morphological and morphometric characteristics of the TMJ across various canine breeds [10]. Although previous investigations have evaluated TMJ morphology, morphometry, and biomechanics, MRI-based assessment of TMJ kinematics has not been reported to date.

Despite the value of static MRI scans for structural assessment, conventional static MRI sequences used in clinical practice may be insufficient for evaluating joint function. Moreover, previous research revealed that static scans do not provide a complete evaluation of the musculoskeletal system dynamics [11,12].

In contrast, dynamic imaging of joints provides a comprehensive assessment of the coordinated interactions among osseous structures, musculature, and surrounding soft tissues that collectively contribute to normal joint function. Consequently, the urgency of developing kMRI techniques for scanning has evolved, and dynamic MRI sequences have been developed for quantification of functional joint movement since the early 90s [13] to understand and differentiate between normal musculoskeletal movements and impaired functions of various joints [14].

kMRI techniques in human medicine can be broadly divided into four categories: incremental positioning, cine-cyclic, active-movement/resistance-based, and real-time imaging. These approaches differ primarily in the manner by which joint motion is generated and recorded, ranging from static assessment at predetermined joint positions to continuous real-time visualization of movement. Collectively, they provide valuable information regarding joint kinematics, biomechanics, and functional behavior under both physiological and stress-loading conditions [15,16,17].

Although kMRI techniques were introduced earlier in human medicine, their application in veterinary medicine has recently been explored. Initial studies focused on assessing the feasibility and clinical utility of kMRI in canine patients. For example, ref. [18] investigated the use of kMRI for the detection of disc-associated cervical spondylomyelopathy in Doberman Pinschers. Additionally, ref. [19] evaluated the feasibility of applying this technique to the cervical spine, elbow, and stifle joints. More recently, ref. [20] demonstrated the value of kMRI in the diagnosis of medial glenohumeral ligament rupture. These studies highlight the growing interest in kMRI as a diagnostic tool for the assessment of musculoskeletal and spinal disorders in veterinary medicine.

Recently observed feline TMJ magnetic resonance and kinematics have not been systematically investigated. Therefore, this research aims to: (1) provide a comprehensive illustration of feline TMJ cross-sectional anatomy with correlation to static MRI scans, (2) assess the joint with kinematic techniques using the real-time kinematic method in intact joints and after manual dislocation of joints in cadavers, (3) evaluate the potential capabilities of MRI to detect TMJ dislocation using static and kinematic scans, (4) quantify and provide statistical justification of TMJ inclination angles at different mouth positions in intact and dislocated status.

## 2. Materials and Methods

### 2.1. Cadaver Selection

Fifteen adult domestic short-haired feline cadavers of both sexes were included in the study. Their body weights ranged from 3.5 to 5 kg, and ages ranged from 1 to 4 years; their causes of death were unrelated to TMJ injuries except for cadaver No. 10, which had a left temporal bone fracture with TMJ dislocation, reflecting a naturally occurring dislocation.

Three frozen cadavers were positioned in sternal recumbency with the head and neck extended. Thereafter, the specimens were serially sectioned at 1 cm intervals using a regular band saw, starting at the level of the nostril tip. Sections demonstrating the TMJ were selected, preserved in 10% formalin, cleaned of debris, and photographed for documentation of normal TMJ anatomy. Structures of the selected sections were subsequently identified and labelled, and the nomenclature was adopted according to [21]. The remaining frozen cadavers were thawed 2–3 days before the scan at room temperature (20 °C) to ensure complete defrosting and softening of all bones, muscles, tendons, and ligaments involved in the TMJ area during the kinematic scan.

### 2.2. Static MRI Protocol

Magnetic resonance data were obtained using an Esaote Vetscan Grande scanner operating at 0.25 T and equipped with a Coil 4 (Esaote S.PA, Genova, Italy). Notably, low-field magnetic resonance machines are mostly used in veterinary practice due to their lower cost and greater accessibility than high-field systems.

A total of 12 cadavers with intact joints were chosen for the static and kinematic MRI scans. For all MRI scans, cadavers were positioned in sternal recumbency with the head extended within the coil, with the support of non-magnetic foam positioning aids to achieve accurate alignment of the TMJ region. Static images were acquired with the mouth in the closed position and then repeated at MIO using a plastic spacer placed between the maxillary and mandibular canine teeth. These plastic spacers were prepared as hollow cylinders of various lengths to accommodate individual cadaver anatomy.

According to the established scanning alignment, TMJ images were acquired in three planes: (1) a sagittal plane perpendicular to the TMJ, (2) a sagittal oblique plane parallel to the mandibular ramus, and (3) a transverse oblique plane parallel to the mandibular ramus. Sagittal and transverse images were obtained using spin-echo T1-weighted (SE T1W) and fast spin-echo T2-weighted (FSE T2W) sequences. These images were used to identify the normal imaging characteristics of the TMJ structures.

For the SE T1W sequence, acquisition parameters were TR = 320 ms, TE = 18 ms, slice thickness = 3 mm, and FOV = 190 × 190 mm. For the FSE T2W sequence, parameters were TR = 2410 ms, TE = 100 ms, slice thickness = 3 mm, and FOV = 180 × 180 mm.

In addition, three-dimensional imaging was performed using a 3D HYCE sequence with acquisition parameters of TR = 10 ms, TE = 5 ms, slice thickness = 0.41 mm, and FOV = 209 × 209 mm, whereas the 3D SST1 sequence acquisition parameters were TR = 30 ms, TE = 15 ms, slice thickness = 0.37 mm, and FOV = 190 × 190 mm. These isotropic sequences enabled multiplanar post-processing reconstruction and detailed evaluation of the joint in all imaging planes.

### 2.3. kMRI Protocol

For kinematic assessment, real-time kMRI images were acquired using a 2D HYCE S sequence, a dedicated kMRI sequence designed for rapid image acquisition during movement. During scanning, the TMJ was manually mobilized by the same operator using a controlled bimanual technique to stabilize the maxilla and mandible, thereby minimizing variability. All kMRI examinations were performed by a board-certified radiologist using a standardized imaging protocol and consistent specimen positioning. Throughout the 60–90 s scan period, the mandible was repeatedly moved smoothly from the closed-mouth position to MIO and back to evaluate the dynamic relationships between the articular surfaces during mandibular movement.

Following image acquisition, the resulting video clips were reviewed and analyzed, and the findings were compared with those obtained from the static MRI scans.

### 2.4. TMJ Dislocation Protocol

A subset of cadavers (numbers 9–12) was subjected to the previously described static and kMRI protocols. Subsequently, after baseline imaging, experimental lateral TMJ dislocation was induced through a standardized anatomical dissection procedure designed to create joint destabilization. To induce dislocation, a skin incision was made over the parotid region, followed by complete removal of the parotid gland to expose the underlying structures. The superficial and deep portions of the masseter muscle were subsequently resected near their origin from the zygomatic arch. The lateral aspect of the TMJ capsule, including the lateral ligament, was then resected to destabilize the joint, after which lateral displacement of the mandibular condyle was manually performed to achieve TMJ dislocation. This dissection protocol was applied to all cadavers of the subgroup and followed by a second MRI scan to evaluate the altered joint configuration to quantify changes in joint angular inclination resulting from TMJ dislocation in both the closed-mouth and MIO positions.

### 2.5. Inclination Angle Measurement

MRI scans were evaluated by radiology personnel included in the study and supervised by a European College of Veterinary Diagnostic Imaging (ECVDI) diplomate. For image analysis, all MRI sequences from the previous scans were subsequently transferred as DICOM files and reviewed using Horos 4.0.1 (Horos Project, Annapolis, MD, USA), a free and open-source DICOM medical image viewer for macOS.

For the static scans, T1W and T2W signal intensities were evaluated in the different TMJ structures. In addition, inclination angles of the right and left TMJs were measured on transverse images obtained with the mouth open and closed in both intact and dislocated joints. These measurements were performed using two reference lines: one parallel to the mandibular fossa of the temporal bone and the other parallel to the mandibular ramus. The inclination angle was defined as the angle formed at the intersection of these two lines. All angle measurements were performed by a single trained observer.

The measurements of the 8 intact TMJ cadavers and the angular measurements after joint dislocation (4 cadavers) were acquired using a standardized protocol to facilitate comparison between intact and dislocated TMJ states, as shown in Table 1. In addition, the post-dislocation subgroup changes in measurements of the last four cadavers are shown in Table 2. All the data were collected for subsequent statistical analysis.

### 2.6. Statistics

Linear mixed-effects models (LMMs) were used to account for the repeated measurements obtained from the same subject and the resulting within-subject correlation. Analyses were performed using R (v4.4.3). For intact joints (*n* = 12), Side, Mouth Position, and their interaction were specified as fixed effects, with Cadaver ID included as a random intercept to account for the correlation among repeated measurements obtained from the same cadaver. For the dislocation subgroup (*n* = 4), Dislocation Status, Side, and Mouth Position and their two- and three-way interactions were specified as fixed effects, with Cadaver ID included as a random intercept to account for the paired pre- and post-dislocation measurements obtained from the same cadaver.

Satterthwaite’s method was used to approximate the denominator degrees of freedom for the omnibus tests. Estimated marginal means were used for post-hoc pairwise comparisons, with Kenward–Roger adjustment of the degrees of freedom. Model assumptions were assessed by visual inspection of residual-versus-fitted plots and normal Q-Q plots, and model singularity was assessed for both models. The diagnostic plots did not indicate major departures from normality or homoscedasticity, although minor deviations from the reference line were observed in the tails of the Q-Q plots. Given the limited number of independent subjects, particularly in the dislocation subgroup (*n* = 4), the dislocation analysis was considered exploratory, and its findings were interpreted accordingly.

Partial eta-squared (*η_p_*^2^) was reported as a measure of effect magnitude and classified as small (≥0.01), medium (≥0.06), or large (≥0.14). Statistical significance was defined as *p* ≤ 0.05. Analyses were conducted using the lme4 (version 2.0-6), lmerTest (version 3.2-1), emmeans (version 2.0.4), and effectsize packages (version 1.0.3).

## 3. Results

Overall, 48 MRI examinations were performed and analyzed, comprising 24 static scans (open- and closed-mouth positions), 12 real-time kinematic scans of intact TMJs, 8 static scans obtained after experimental dislocation, and 4 post-dislocation real-time kinematic scans. In cadavers 1 to 8, the total imaging and evaluation time ranged from 2 to 3 h before joint dislocation and increased to 3 to 4 h following induction of the dislocation.

### 3.1. Anatomical Assessment of Joint Structures in Correlation with Magnetic Resonance

**Figure 1:**
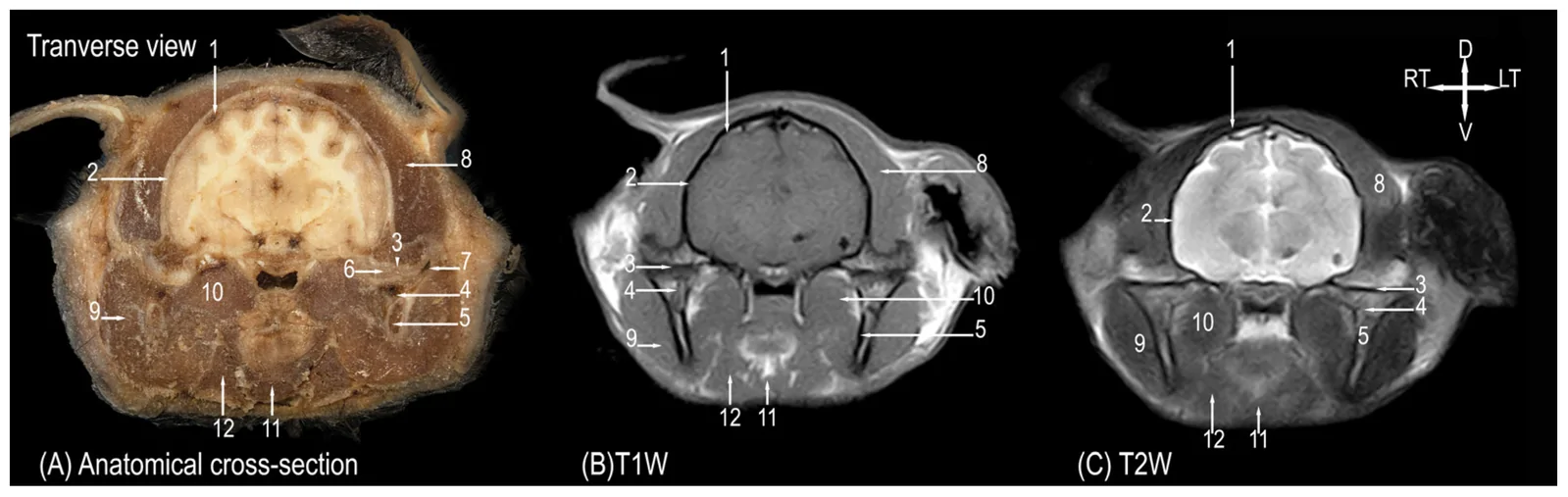
Correlation of cross-sectional anatomy with transverse MRI images obtained in the closed-mouth position at the level of the TMJ in feline cadaver no. 12. (**A**) Anatomical cross-sectional reference image. (**B**) Transverse T1W MRI image. (**C**) Transverse T2-W MRI image. Orientation markers: RT (right), LT (left), D (dorsal), and V (ventral). Labeled anatomical structures (1. Os parietale, 2. Os temporale, 3. Fossa mandibularis, 4. Processus condylaris, 5. Ramus mandibulae, 6. Discus articularis, 7. Lig. Laterale, 8. M. Temporalis, 9. M. Masseter, 10. M. Pterygoideus medialis, 11. M. Mylohyoideus, 12. M. Digastricus). The figure demonstrates the normal osseous and soft-tissue structures of the TMJ, providing an anatomical basis for MRI interpretation and clinical assessment of TMJ abnormalities in cats.

Anatomically, the cross-section of the feline TMJ (Figure 1A) revealed the rostral portion of the parietal bone (1) dorsally and the squamous part of the temporal bone (2) laterally. The articular components of the TMJ consisted of the mandibular fossa of the temporal bone (3), the condylar process (4) of the mandibular ramus (5), and an intervening articular disc (6), composed of a thin layer of fibrocartilage between the opposing articular surfaces, contributing to joint congruency. The joint capsule was laterally reinforced by a dense fibrous thickening that formed the lateral ligament (7).

The principal masticatory muscles associated with the TMJ and visible in this section were the temporalis muscle (8), masseter muscle (9), medial pterygoid muscle (10), and digastric muscle (12).

On MRI scans (Figure 1B,C), the osseous components of the TMJ exhibited central hyperintensity corresponding to fatty bone marrow, surrounded by a peripheral hypointense cortical bone layer on both T1W and T2W sequences.

The masticatory muscles appeared hypointense relative to the temporal bone and mandibular condyle, whereas adipose tissue displayed marked hyperintensity on T1W images and ranged from iso- to hyperintense on T2W sequences.

The articular disc, synovial fluid, and lateral ligament could not be distinctly visualized on either sequence.

### 3.2. Inclination Angle Measurements of the Right and Left TMJs in MIO and Closed-Mouth Positions in Intact and Dislocated Joints (Figure 2)

To facilitate comparison between pre- and post-dislocation measurements, Table 2 summarizes the changes in TMJ angles. Post-dislocation values demonstrated an increase across all specimens and measurement conditions. The greatest mean increase was observed in the right TMJ during MIO (+2.65°), whereas the smallest change was observed in the left TMJ in the closed-mouth position (+1.48°).

**Figure 2:**
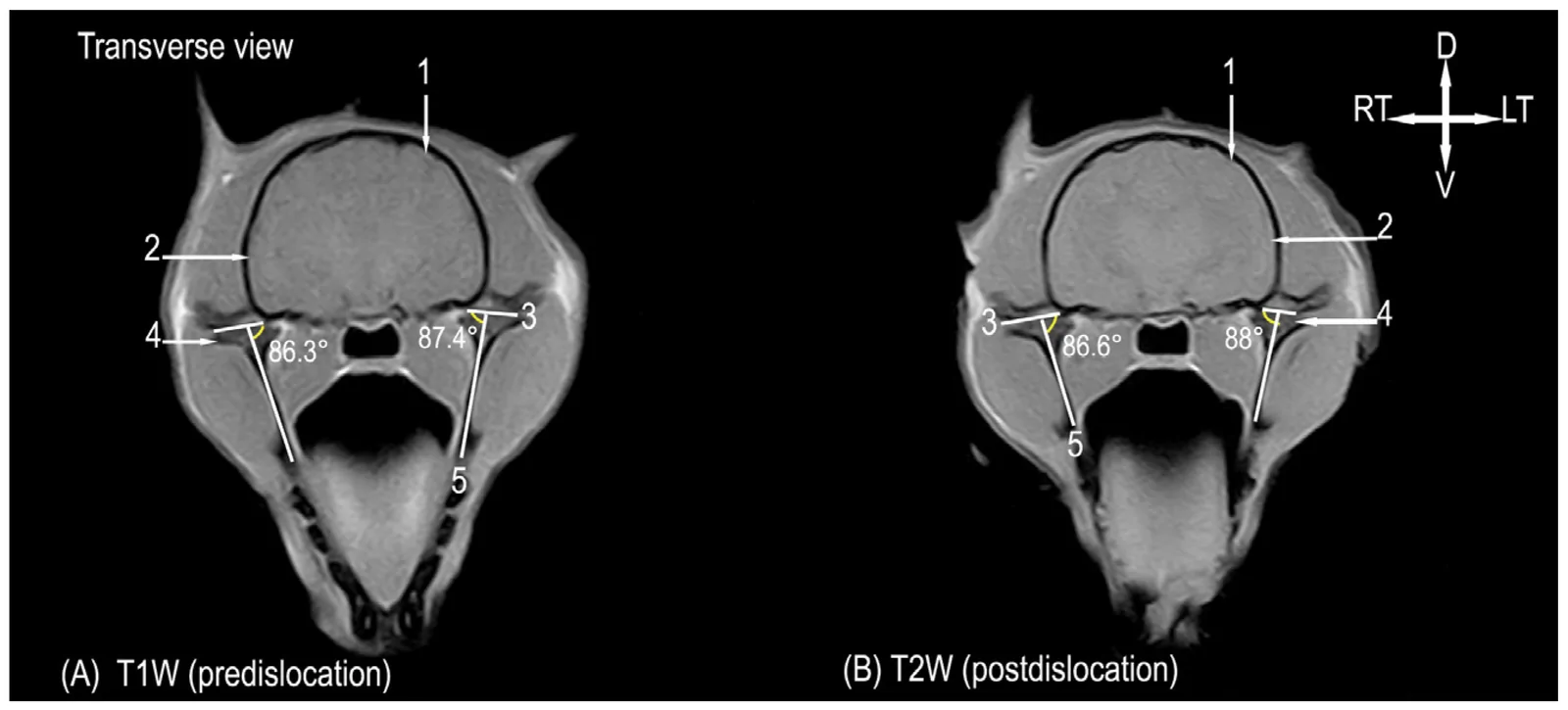
Transverse T1W static MRI images at the level of the TMJs in the MIO of feline cadaver no. 11. (**A**) Pre-manual dislocation. (**B**) Post-manual dislocation. Orientation markers: RT (right) and LT (left), D (dorsal), and V (ventral). Labeled anatomical structures (1. Os parietale, 2. Os temporale, 3. Fossa mandibularis, 4. Processus condylaris, 5. Ramus mandibulae). The inclination angle, marked in yellow, intersected between lines 3 and 5. The images illustrate changes in TMJ alignment associated with manual dislocation and demonstrate the utility of MRI-based inclination angular measurements for assessing TMJ morphology and joint mechanics.

### 3.3. Comparative Evaluation of Intact and Dislocated TMJs in Static Scans Using 3D Sequences in the Closed-Mouth Position

**Figure 3:**
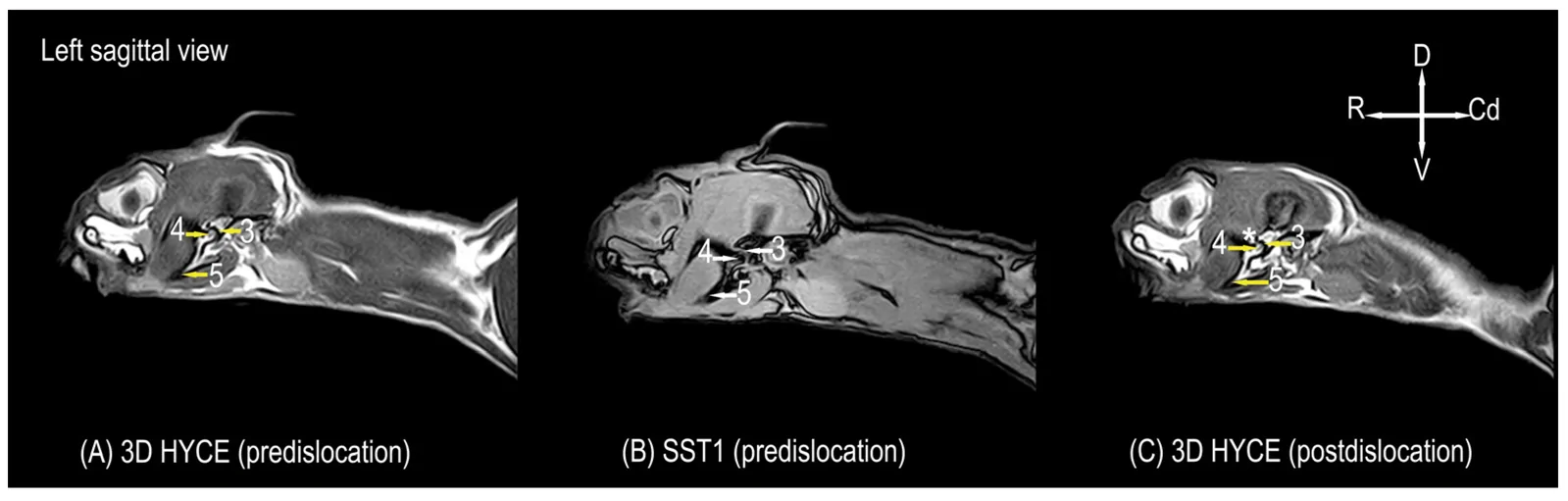
Sagittal 3D HYCE and SST1 static MRI images of the left TMJ in feline cadaver no. 12 in the closed-mouth position. (**A**) Pre-dislocation 3D HYCE image. (**B**) Pre-dislocation SST1 image. (**C**) Post-dislocation 3D HYCE image (asterisk). Orientation markers: R (rostral) and Cd (caudal), D (dorsal), and V (ventral). Labeled anatomical structures (3. Fossa mandibularis, 4. Processus condylaris, 5. Ramus mandibulae). The images demonstrate MRI’s ability to identify TMJ dislocation in the closed-mouth position and to visualize associated changes in joint alignment and condylar position.

The 3D HYCE and SST1 sequences (Figure 3A,B) provided complementary visualization of the TMJ structures. On the 3D HYCE sequence, the osseous structures appeared hyperintense relative to the adjacent musculature, with a sharply defined hypointense cortical margin, while remaining hypointense relative to fat and fluid-containing tissues.

In contrast, the SST1 sequence depicted the osseous components as hypo- to isointense relative to muscle, with fluid-containing structures exhibiting low signal intensity. Notably, the SST1 sequence (Figure 3B) provided superior delineation of the joint space, allowing more precise visualization of the articular interface and improving anatomical interpretation of the TMJ.

Static MRI scans acquired with 3D sequences in the closed-mouth position (Figure 3) demonstrated no visually appreciable differences between intact (Figure 3A,B) and dislocated (Figure 3C) TMJ on qualitative assessment. However, quantitative analysis of inclination angles in the transverse plane revealed subtle alterations between pre-dislocation and post-dislocation measurements in both TMJs.

### 3.4. Comparison of Intact and Dislocated TMJs on Static MRI in the MIO Position

**Figure 4:**
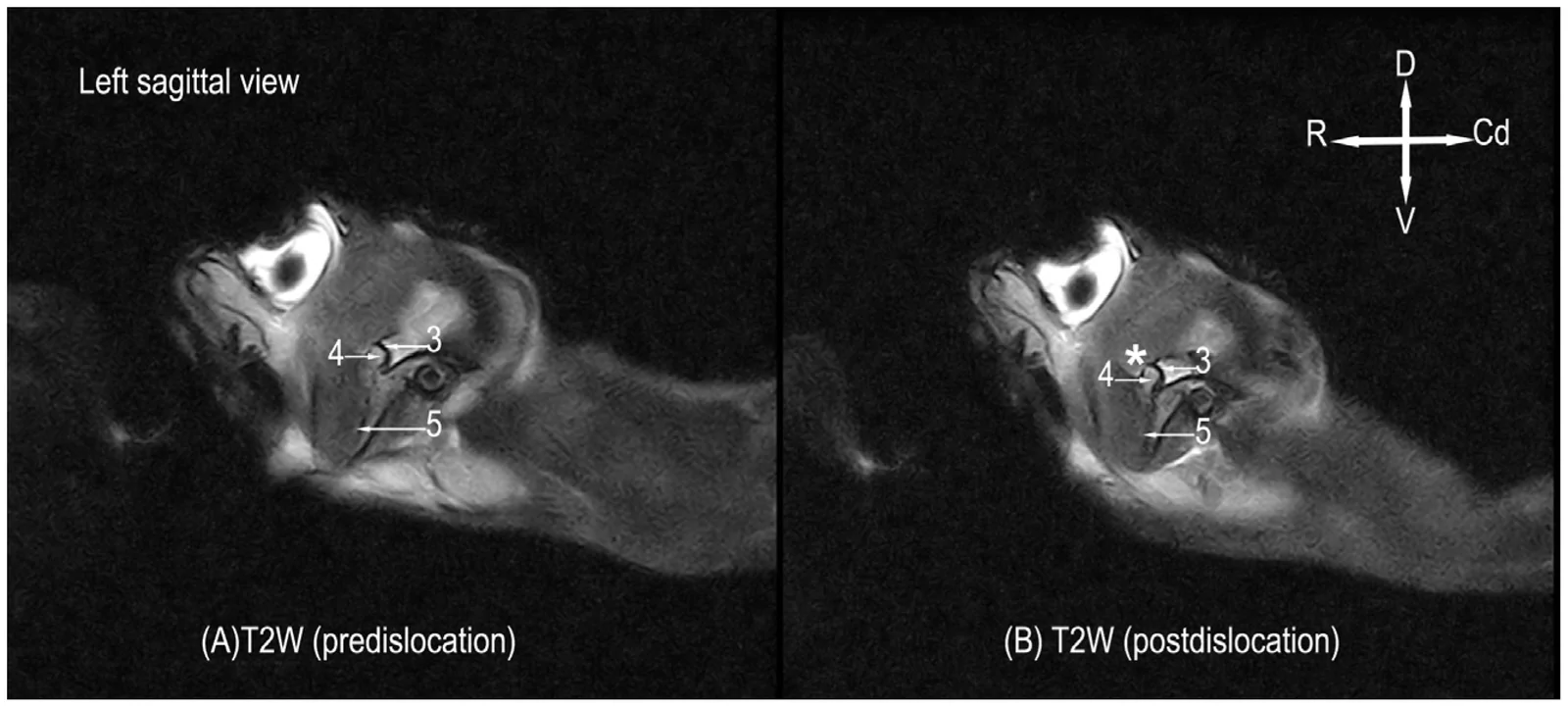
Sagittal T2W static MRI images of the left TMJ in feline cadaver no. 12 in the MIO position. (**A**) Pre-dislocation, with the mandibular condyloid process in its normal anatomical position. (**B**) Post-dislocation, showing rostral displacement of the mandibular condyloid process (asterisk). Orientation markers: R (rostral) and Cd (caudal), D (dorsal), and V (ventral). Labeled anatomical structures (3. Fossa mandibularis, 4. Processus condylaris, 5. Ramus mandibulae). The images demonstrate MRI-detectable changes in TMJ alignment following dislocation and illustrate the utility of MRI for evaluating condylar position and joint integrity.

In contrast to the static closed-mouth scans (Figure 3), static MRI acquired at MIO (Figure 4) demonstrated evident rostral luxation of the mandibular condylar process on sagittal oblique acquisition. This finding was accompanied by a moderate increase in the transverse inclination angles of both the right and left TMJs, indicating altered condylar orientation following mouth opening.

### 3.5. Evaluation of a Dislocated Joint Due to Left Temporal Bone Fracture

**Figure 5:**
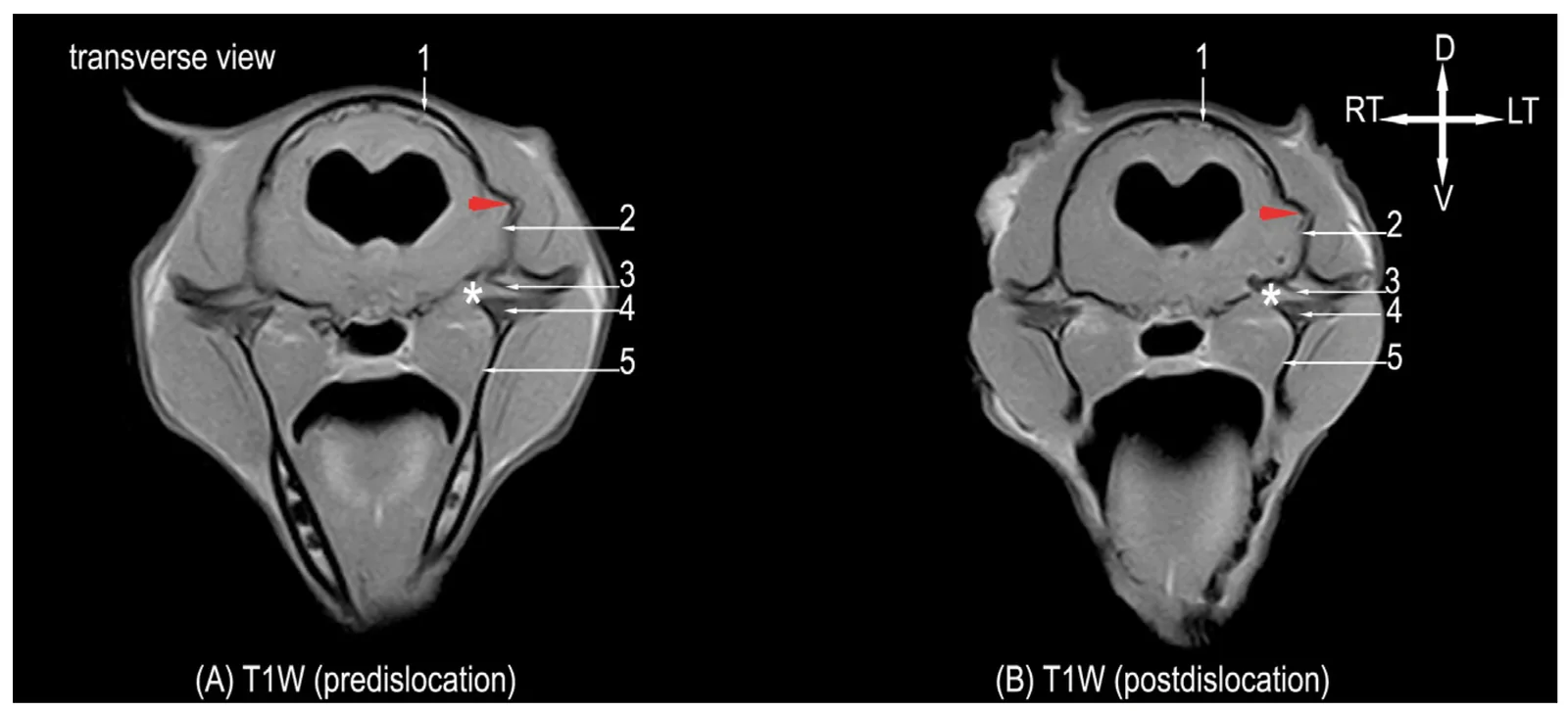
T1W static MRI images of feline cadaver no. 10 in the open-mouth position. (**A**) Pre-dislocation. (**B**) Post-dislocation. Orientation markers: RT (right) and LT (left), D (dorsal), and V (ventral). Labeled anatomical structures (1. Os parietale, 2. Os temporale, 3. Fossa mandibularis, 4. Processus condylaris, 5. Ramus mandibulae). TMJ dislocation caused by a left temporal bone fracture is indicated by an asterisk in (**A**,**B**). The temporal bone fracture is indicated by a red arrowhead in (**A**,**B**). The images demonstrate MRI-detectable changes in TMJ morphology and alignment secondary to temporal bone fracture-induced dislocation, highlighting the utility of MRI for identifying associated fractures and assessing joint instability.

A temporal bone fracture resulted in marked TMJ instability, characterized by substantial widening of the left TMJ inclination angle (Figure 5). This joint exhibited the greatest inclination values observed in the study compared with intact joints (Table 1), measuring 84.9° in the closed-mouth position and 87.9° during mouth opening. In addition, incongruity between the mandibular condyle and the articular surface was evident, creating a pronounced gap in the inclination angle and indicating loss of normal joint congruence. These findings suggest that the increased inclination angles were associated with clinically relevant alterations in joint morphology and stability rather than isolated numerical differences. In contrast, experimentally induced dislocation of the contralateral (right) TMJ produced no appreciable morphological alterations.

### 3.6. Real-Time kMRI

**Figure 6:**
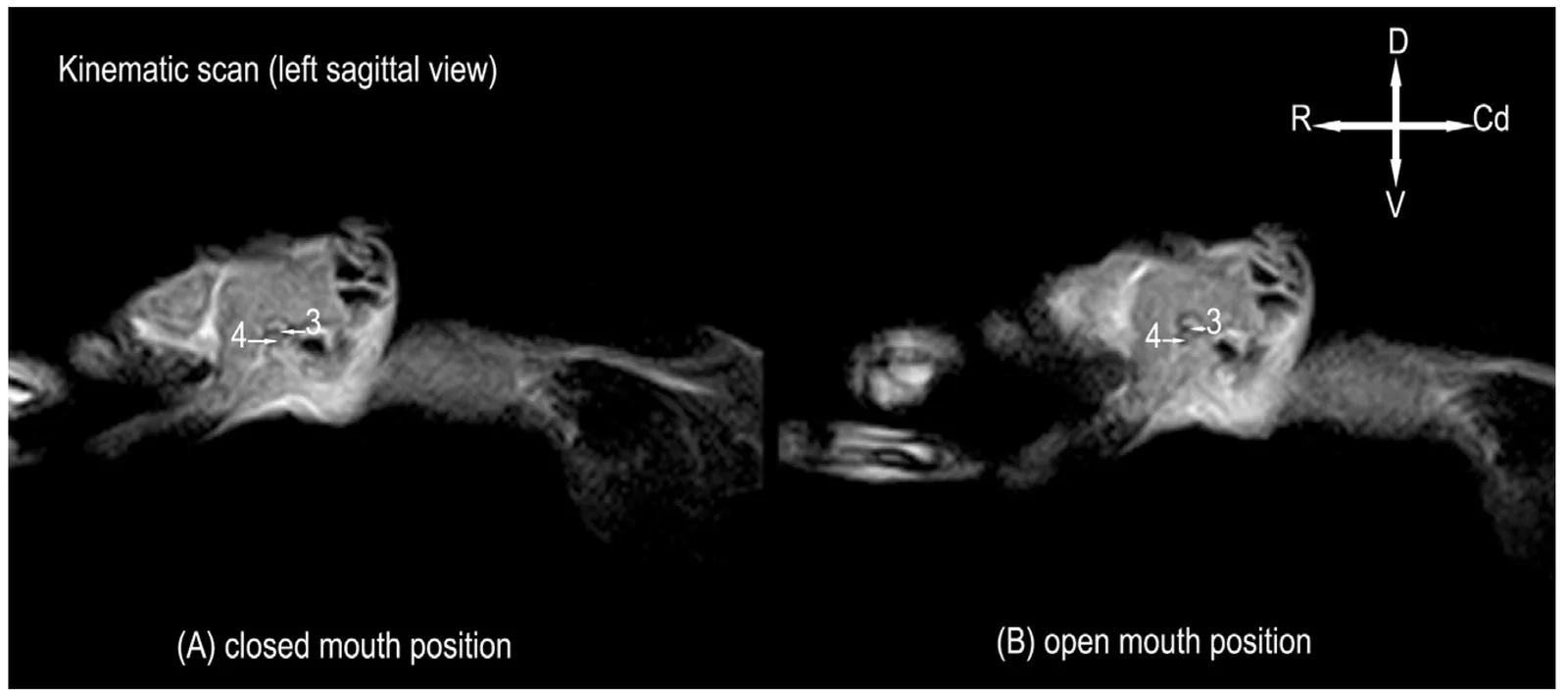
Sagittal oblique real-time kMRI images of the TMJ in feline cadaver no. 2. (**A**) Closed-mouth position. (**B**) Open-mouth position. Orientation markers: R (rostral) and Cd (caudal), D (dorsal), and V (ventral). Labeled anatomical structures (3. Fossa mandibularis, 4. Processus condylaris). The images were acquired to assess the feasibility of real-time kMRI for dynamic evaluation of TMJ motion.

Collectively, the evaluation of the kinematic real-time scans (Figure 6, Supplementary Material Video S1) in both the sagittal and transverse planes was rated as poor to unacceptable across all cadavers. Although each scan lasted approximately 60–90 s and was performed using slow, repeated step-by-step movements of the jaws, image quality was substantially degraded by motion artifacts.

Consequently, despite the potential value of kMRI for evaluating dynamic joint function, the current implementation was insufficiently developed to provide reliable diagnostic information.

### 3.7. Statistical Analysis of TMJ Inclination Angles

#### 3.7.1. Inclination Angle Analysis via Linear Mixed-Effects Models (Figure 2)

For intact joints (Table 3), the linear mixed-effects model identified significant main effects of Side (*F*(1, 33) = 10.74, *p* = 0.002, *η_p_*^2^ = 0.25) and Mouth Position (*F*(1, 33) = 83.73, *p* < 0.001, *η_p_*^2^ = 0.72). These findings indicate that TMJ inclination angles differed between the anatomical sides and between closed and open mouth positions. The Side × Mouth Position interaction was not statistically significant (*F*(1, 33) = 0.22, *p* = 0.642, *η_p_*^2^ < 0.01), indicating no evidence that the side-related difference in inclination angle varied significantly according to mouth position.

#### 3.7.2. Post-Hoc Pairwise Comparisons (Table 4)

The analysis showed that the estimated Left-Right difference was 1.49° in the closed-mouth position and 1.99° in the open-mouth position. The difference was statistically significant in the open-mouth position (mean difference = 1.99°, SE = 0.751°, t(33) = 2.65, *p* = 0.012), whereas the corresponding comparison in the closed-mouth position did not reach the predefined significance threshold (mean difference = 1.49°, SE = 0.751°, t(33) = 1.99, *p* = 0.055).

**Table 4:** Post-hoc pairwise comparisons of TMJ inclination angles between the left and right sides at each mouth position.

Mouth Position	Contrast	Estimate	SE	df	*t*-Ratio	*p*-Value
Closed	LT–RT	1.49	0.751	33	1.99	0.056
Open	LT–RT	1.99	0.751	33	2.65	0.012

LT = left side; RT = right side; SE = standard error; df = degrees of freedom. Pairwise comparisons were based on estimated marginal means, with Kenward–Roger adjustment of the degrees of freedom. Statistical significance was defined as *p* ≤ 0.05.

#### 3.7.3. Exploratory Analysis of Dislocated Joints Subgroup

Within the subgroup subjected to experimental dislocation (*n* = 4), the mixed-effects model identified significant main effects of Dislocation Status, Side, and Mouth Position (Table 5). Dislocation Status was significantly associated with TMJ inclination angles (*F*(1, 21) = 10.04, *p* = 0.005, *η_p_*^2^ = 0.32). Significant effects were also observed for Side (*F*(1, 21) = 18.13, *p* < 0.001, *η_p_*^2^ = 0.46) and Mouth Position (*F*(1, 21) = 33.18, *p* < 0.001, *ηp*^2^ = 0.61). None of the two-way or three-way interactions reached statistical significance (*p* > 0.05). Given the limited number of independent subjects in the dislocation subgroup (*n* = 4), this analysis was considered exploratory.

## 4. Discussion

Cross-sectional anatomical examination of the feline TMJ demonstrated both the osseous and soft-tissue components of the joint. Among the structures identified, the articular disc appeared as a thin fibrocartilaginous structure, while the joint capsule was reinforced laterally by the lateral ligament. These findings are consistent with those reported by [9], who described lateral reinforcement of the feline TMJ capsule following detailed microdissection. Furthermore, the articular disc was attached circumferentially to the joint capsule, thereby dividing the joint cavity into separate dorsal and ventral compartments.

Despite the anatomical identification of these structures, the articular disc, lateral ligament, and synovial fluid could not be clearly visualized on low-field MRI in either the open- or closed-mouth positions. In contrast, ref. [22] reported that visualization of the articular disc in dogs was more feasible in the open-mouth position than in the closed-mouth position using T1W and T2W sequences acquired with a 1.0-T MRI system. Similarly, ref. [23] demonstrated that static MRI provided superior visualization of the TMJ disc compared with dynamic imaging in human subjects. In the present study, the use of an ankle coil yielded high-quality static images. Comparable findings were reported by [24], who evaluated feline TMJs using a low-field MRI system and demonstrated improved image quality with a surface coil compared with a knee coil.

Concerning imaging protocols, static MRI provided superior anatomical detail and more reliable visualization of joint structures than kinematic imaging. Furthermore, rostral displacement of the mandibular condyle in dislocated joints was more readily identified on static images acquired in the MIO than on those obtained in the closed-mouth position.

Although the dislocation protocol involved invasive dissection, the cadaveric model provided a controlled experimental environment that enabled standardized joint destabilization and direct assessment of TMJ biomechanics. This approach minimized the influence of physiological variables present in live animals, such as muscle activity and voluntary motion, allowing consistent evaluation of changes in joint alignment following dislocation. Consequently, the model was considered appropriate for biomechanical inference regarding TMJ stability and kinematics.

Interestingly, experimentally induced dislocations did not demonstrate obvious morphological differences from intact joints on static closed-mouth images, despite measurable differences in TMJ inclination angles. This finding suggests that quantitative angular measurements may detect subtle alterations in joint orientation that are not readily apparent during qualitative image assessment alone. Consequently, objective morphometric analyses may provide additional diagnostic value in the evaluation of TMJ instability.

The morphological findings further indicated that TMJ inclination angles were more profoundly affected by disruption of the temporal bone and the associated loss of joint congruency, characterized by a marked separation between the mandibular condyle and the articular surface, than by experimental dislocation alone. These observations emphasize that inclination-angle measurements should be interpreted alongside imaging findings, as statistically measurable differences may not necessarily correspond to clinically relevant structural alterations.

Quantitative analysis demonstrated that mouth position was the primary determinant of TMJ inclination angle, exhibiting the largest effect size among all investigated variables. This finding highlights the dynamic nature of the feline TMJ and indicates that mandibular opening exerts a substantial influence on joint orientation. The increased inclination angles observed during mouth opening likely reflect physiological translation of the mandibular condyle along the articular surface, representing a normal biomechanical adaptation that facilitates mandibular movement. The large associated effect size further suggests that these differences are biologically meaningful in addition to being statistically significant.

Differences between the left and right TMJs were also observed, although their magnitude was smaller than that associated with mouth position. Post hoc analyses revealed that these differences were most evident during mouth opening, suggesting that functional loading may accentuate subtle anatomical or biomechanical asymmetries between the two joints. Conversely, the absence of significant side-related differences in the closed-mouth position suggests that such asymmetries become apparent primarily during movement. The confidence intervals associated with the calculated effect sizes further support the reliability of these findings despite the relatively limited sample size.

Experimentally induced dislocation significantly altered TMJ inclination angles and was associated with a large effect size, indicating that condylar displacement substantially influences overall joint orientation. Notably, no significant interaction effects were detected among dislocation status, side, and mouth position, suggesting that these variables exerted largely independent effects on joint orientation.

The detected variations in TMJ inclination angles imply that dislocation leads to changes in joint alignment and could be used as a quantitative marker of structural displacement and joint instability. However, further investigations in living patients are needed to determine their clinical applicability and define diagnostic threshold values.

Although all measurements were performed according to a standardized protocol, formal assessments of intra-observer repeatability and inter-observer agreement were not conducted. Therefore, the reproducibility and reliability of the measurements could not be objectively quantified. This represents a limitation of the study and may have influenced measurement consistency. In addition, the relatively small number of specimens included in the dislocation subgroup. This limitation may have reduced the statistical power of the analyses and restricted the generalizability of the findings.

Several alternative approaches have been described for the diagnosis of TMJ dislocation. These include oblique radiography [25] and assessment of the vertical mandibular range of motion, which permits measurement of MIO in anesthetized cats with healthy TMJs [1]. Additionally, ref. [26] evaluated gape angles by measuring the interincisal distance in conscious and anesthetized cats and concluded that gape-angle measurements are not reliable indicators of oral pain.

kMRI was originally developed to evaluate pathological joint conditions that are position-dependent or influenced by functional loading and biomechanical stress [27]. However, in the present study using cadaveric specimens, the diagnostic utility of real-time kMRI performed by a low-field open-bore MRI system (0.25 T) was limited for the reliable assessment of feline TMJ dislocation due to reduced SNR, resulting in decreased image quality, prolonged acquisition times, lower spatial resolution, and suboptimal soft-tissue contrast [28], as well as motion artifacts.

The use of thawed cadaveric specimens may have further influenced the biomechanical behavior of the joint and surrounding soft tissues. Previous investigations demonstrated that the influence of freezing and thawing on biomechanical properties varies among tissue types; for instance, musculoskeletal tissues may exhibit altered mechanical behavior following cycles [29], while articular cartilage stored at −20 °C and rapidly thawed has been reported to maintain mechanical stiffness comparable to non-frozen controls [30].

Therefore, although all specimens in the current study were subjected to the same preservation protocol, the potential impact of thawing on joint biomechanics and kMRI findings cannot be completely excluded.

As a result, the overall diagnostic performance of real-time kMRI for detecting feline TMJ dislocation was restricted in this study. Therefore, future investigations employing high-field MRI systems are recommended to improve image quality and facilitate a more comprehensive assessment of feline TMJ dynamics.

These findings are supported by the observations of [31], who evaluated joint kinematics using both 0.5-T open-bore and 1.5-T MRI systems and reported fewer motion artifacts, reduced image blurring, and improved measurement accuracy with the higher-field scanner. Moreover, measurements obtained with the open-bore system were less accurate than those acquired using the 1.5-T MRI unit. Similarly, ref. [23] reported that static MRI was superior to dynamic MRI for visualizing the condylar head. In agreement with these observations, ref. [19] demonstrated that motion artifacts were initially present during real-time kMRI evaluation of the canine elbow but decreased substantially following operator training and procedural refinement.

Conversely, ref. [32] reported that high-field real-time kMRI provides valuable information regarding human TMJ disc and condylar mobility, including visualization of rostral and caudal disc reduction and assessment of dynamic changes in the disc-condyle relationship throughout the entire range of mandibular motion. Nevertheless, ref. [33] concluded that dynamic MRI cannot replace conventional static MRI in the evaluation of internal TMJ derangements. Rather, dynamic imaging should be regarded as a complementary technique that augments the diagnostic information obtained from routine static MRI examinations.

## 5. Conclusions

Based on our results in this cadaveric model, static MRI was a feasible technique for detecting feline TMJ dislocation. Morphological detection of dislocation was highly dependent on mouth position, with the open-mouth position providing the clearest visualization of the dislocated joint. However, quantitative measurements of TMJ inclination angles were able to identify dislocation-related changes even in the closed-mouth position, where morphological alterations were less apparent. Although kMRI has the potential to provide additional information regarding TMJ motion, its application under the current low-field imaging conditions was limited. Given the limited number of dislocation cases and the predominance of experimentally induced dislocations, these findings should be interpreted as evidence of feasibility under controlled conditions. Further studies involving larger sample sizes, naturally occurring cases, and in vivo investigations are needed to determine whether these observations translate to clinical patients and to evaluate the potential clinical value of these imaging approaches.

## Figures and Tables

**Table 1:** Inclination angle measurements of the right and left TMJs in closed- and open-mouth positions for intact joints (*n* = 8 cadavers) and the pre- and post-dislocation subgroup (*n* = 4 cadavers).

Cadaver	Closed Mouth		(MIO)	
	RT-TMJ	LT-TMJ	RT-TMJ	LT-TMJ
1st	72.7°	73.5°	79.5°	80°
2nd	68.7°	70.6°	79.5°	75.5°
3rd	76.4°	78.7°	78°	85.3°
4th	71.5°	72.2°	78.1°	80.3°
5th	78.8°	80.2°	82.9°	83.43°
6th	68.4°	68.9°	71.35°	74.1°
7th	81.4°	78°	86.5°	87.9°
8th	82.7°	83.2°	85.6°	89.6°
9th (pre-dislocation)	73.4°	78.7°	77.5°	80.7°
9th (post-dislocation)	74.5°	80.4°	85°	84.7°
10th (pre-dislocation)	79.5°	84.9°	83.5°	87.9°
10th (post-dislocation)	81°	86.6°	84.5°	89.6°
11th (pre-dislocation)	82.5°	84°	86.3°	87.4°
11th (post-dislocation)	84.5°	85.8°	86.6°	88.1°
12th (pre-dislocation)	80.7°	81.7°	83.3°	83.8°
12th (post-dislocation)	82.6°	82.4°	85.1°	85.2°

**Table 2:** Changes in TMJ inclination angles following dislocation (post-dislocation minus pre-dislocation).

Cadaver	RT-TMJ (Closed Mouth)	LT-TMJ (Closed Mouth)	RT-TMJ (MIO)	LT-TMJ (MIO)
9th	+1.1°	+1.7°	+7.5°	+4.0°
10th	+1.5°	+1.7°	+1.0°	+1.7°
11th	+2.0°	+1.8°	+0.3°	+0.7°
12th	+1.9°	+0.7°	+1.8°	+1.4°
Mean change	+1.63°	+1.48°	+2.65°	+1.95°

**Table 3:** Linear Mixed-effects model ANOVA and effect sizes for intact TMJ inclination angles.

Predictor 95.	Sum Sq.	Mean Sq.	Num. df	Den. df	*F*-Value	*p*-Value	Partial *η_p_*^2^
Side (RT vs. LT)	36.37	36.37	1	33	10.74	0.002	0.25
Mouth Position (Closed vs. Open)	283.63	283.63	1	33	83.73	<0.001	0.72
Side × Mouth Position	0.75	0.75	1	33	0.22	0.642	<0.01

Model fit using a linear mixed-effects model. Sum Sq. = sum of squares; Mean Sq. = mean squares; Num. df = numerator degrees of freedom; Den. df = denominator degrees of freedom, calculated using Satterthwaite’s method. Statistical significance was defined as *p* ≤ 0.05. Partial eta-squared (*η_p_*^2^) was reported as a measure of effect magnitude and classified as small (≥0.01), medium (≥0.06), and large (≥0.14). LT = left side; RT = right side.

**Table 5:** Linear mixed-effects model ANOVA and effect sizes for pre- versus post-dislocation conditions.

Predictor/Effect	Sum Sq.	Mean Sq.	Num. df	Den. df	*F*-Value	*p*-Value	Partial *η_p_*^2^
Dislocation Status	29.65	29.65	1	21	10.04	0.005	0.32
Side	53.56	53.56	1	21	18.13	<0.001	0.46
Mouth Position	98.00	98.00	1	21	33.18	<0.001	0.61
Dislocation × Side	0.36	0.36	1	21	0.12	0.730	<0.01
Dislocation × Mouth	1.13	1.13	1	21	0.38	0.544	0.02
Side × Mouth	3.25	3.25	1	21	1.10	0.306	0.05
Dislocation × Side × Mouth	0.15	0.15	1	21	0.05	0.823	<0.01

Model fit using a linear mixed-effects model. Sum Sq. = sum of squares; Mean Sq. = mean squares; Num. df = numerator degrees of freedom; Den. df = denominator degrees of freedom, calculated using Satterthwaite’s method. Statistical significance was defined as *p* ≤ 0.05. Partial eta-squared (*η_p_*^2^) was reported as a measure of effect magnitude and classified as small (≥0.01), medium (≥0.06), and large (≥0.14).

## Data Availability

The original contributions presented in this study are included in the article. Further inquiries can be directed to the corresponding authors.

## Supplementary Materials

The following supporting information can be downloaded at: https://www.mdpi.com/article/10.3390/vetsci13090956/s1, Video S1: feline TMJ real-time kMRI.
